# Effects of Exogenous Isosteviol on the Physiological Characteristics of *Brassica napus* Seedlings under Salt Stress

**DOI:** 10.3390/plants13020217

**Published:** 2024-01-12

**Authors:** Wenjing Xia, Wangang Meng, Yueqin Peng, Yutian Qin, Liang Zhang, Nianqing Zhu

**Affiliations:** 1 School of Chemistry and Bioengineering, Taizhou College, Nanjing Normal University, Taizhou 225300, China; xiawenjing@nnutc.edu.cn (W.X.); 16210252@nnutc.edu.cn (W.M.);; 2 Jiangsu Key Laboratory of Chiral Pharmaceuticals Biosynthesis, College of Pharmacy and Chemistry & Chemical Engineering, Taizhou University, Taizhou 225300, China

**Keywords:** salt stress, isosteviol, *Brassica napus* seedlings, physiological characteristics

## Abstract

In this paper, the effect of isosteviol on the physiological metabolism of *Brassica napus* seedlings under salt stress is explored. *Brassica napus* seeds (Qinyou 2) were used as materials, and the seeds were soaked in different concentrations of isosteviol under salt stress. The fresh weight, dry weight, osmotic substance, absorption and distribution of Na^+^, K^+^, Cl^−^, and the content of reactive oxygen species (ROS) were measured, and these results were combined with the changes shown by Fourier transform infrared spectroscopy (FTIR). The results showed that isosteviol at an appropriate concentration could effectively increase the biomass and soluble protein content of *Brassica napus* seedlings and reduce the contents of proline, glycine betaine, and ROS in the seedlings. Isosteviol reduces the oxidative damage to *Brassica napus* seedlings caused by salt stress by regulating the production of osmotic substances and ROS. In addition, after seed soaking in isosteviol, the Na^+^ content in the shoots of the *Brassica napus* seedlings was always lower than that in the roots, while the opposite was true for the K^+^ content. This indicated that under salt stress the Na^+^ absorbed by the *Brassica napus* seedlings was mainly accumulated in the roots and that less Na^+^ was transported to the shoots, while more of the K^+^ absorbed by the *Brassica napus* seedlings was retained in the leaves. It is speculated that this may be an important mechanism for *Brassica napus* seedlings to relieve Na^+^ toxicity. The spectroscopy analysis showed that, compared with the control group (T1), salt stress increased the absorbance values of carbohydrates, proteins, lipids, nucleic acids, etc., indicating structural damage to the plasma membrane and cell wall. The spectra of the isosteviol seed soaking treatment group were nearly the same as those of the control group (T1). The correlation analysis shows that under salt stress the *Brassica napus* seedling tissues could absorb large amounts of Na^+^ and Cl^−^ to induce oxidative stress and inhibit the growth of the plants. After the seed soaking treatment, isosteviol could significantly reduce the absorption of Na^+^ by the seedling tissues, increase the K^+^ content, and reduce the salt stress damage to the plant seedlings. Therefore, under salt stress, seed soaking with isosteviol at an appropriate concentration (10^−9^~10^−8^ M) can increase the salt resistance of *Brassica napus* seedlings by regulating their physiological and metabolic functions.

## 1. Introduction

Soil salinization is one of the abiotic stresses that affects the growth and development of crops, severely restricting the improvement in crop yield and quality. The area of various types of salinized land in China has exceeded 1 × 10^8^ hm^2^, and it is mainly concentrated in the northern and coastal areas [1,2]. The coastline of the coastal area of Jiangsu Province is 744 km long, and the area is mostly a silt coast. The coastal tidal flat area is approximately 6.53 × 10^5^ hm^2^, ranking first among all provinces and cities. With economic development in China, the increase in industrial pollution, and irrational irrigation and fertilization methods, the area of secondary salinized land continues to increase year by year. In recent years, to accelerate the improvement, development, and utilization of tidal flat salinized land, various types of plants have been introduced and planted in the coastal tidal flats of Jiangsu Province. Among them, rape, as a winter and spring crop, is planted on coastal salinized land and can effectively alleviate the oil and grain supply shortage in China and give full play to the dual benefits of salinized soil productivity [3]. In addition, in a high-salt environment plants accumulate a large amount of Na^+^ in their cells. A high concentration of Na^+^ produces the dual effects of salt toxicity and osmotic damage to the body, leading to an imbalance in reactive oxygen species (ROS) metabolism and a reduction in photosynthetic efficiency [4]. A large amount of ROS accumulated in plants can oxidize and attack the cell membrane system, causing local damage to the plasma membrane, an increase in nonselective permeability, a large amount of membrane leakage of cell contents, and the arrest of plant growth, and, in severe cases, it can have an impact on plant survival [5]. At present, there are a few types of rapeseed varieties that have been selected and bred for salt tolerance; these varieties have weak salt tolerance. Therefore, mitigating the harm to plants caused by soil salinization and enhancing plant salt tolerance will help to improve crop yield and quality and reduce the economic losses caused by salt stress.

The seedling stage is the period in the growth cycle when physiological metabolic function is the most exuberant and sensitive [6,7], and it is very susceptible to the influence of various external environmental factors. In recent years, the application of plant growth regulatory substances in the process of plant seed germination and seedling formation has become the focus of attention, especially in terms of the function and protective mechanism of these substances in plant resistance to salt stress, where greater progress has been made. Ahanger et al. (2020) found that with the combined action of brassinosteroids and kinetin, they could reduce salt stress-induced damage in tomato plants by regulating the metabolic function of antioxidants and osmotic agents [8]. Ahmad et al. (2016) found that the external application of nitric oxide (NO) could increase the biomass and antioxidant enzyme activities of chickpea (*Cicer arietinum* L.) plants under NaCl stress, reduce the production of ROS and malondialdehyde (MDA), and improve plant salt resistance [9]. El Hamdaoui et al. (2021) found that when the NaCl concentration exceeded 150 mM, the *Lavandula mairei* seed germination rate was significantly reduced; however, soaking seeds with gibberellin could increase the germination rate of *Lavandula mairei* seeds under salt stress [10]. In addition, substances such as 6-benzylaminoadenine, ascorbic acid, melatonin, selenium, and sulfur can also exert a good mitigation effect when plants are under salt stress [11,12,13].

Isosteviol is a tetracyclic diterpenoid with a beyerane skeleton that was first reported by Mosetling and Nes in 1955 [14,15]. Recently, studies have shown that isosteviol has biological activities similar to those of gibberellin in alleviating abiotic stress in plant seedlings [16,17]. For example, Wang et al. (2022a) found that soaking seeds in isosteviol (10^−9^~10^−8^ M) could effectively relieve the growth inhibition effect of chromium stress on wheat seedlings, thereby improving the stress tolerance of wheat [18]. However, studies on the effects of exogenous isosteviol on the physiological characteristics of *Brassica napus* seedlings under salt stress are scarce. Therefore, this experiment used *Brassica napus* (Qinyou 2) as the test material to investigate the effects of soaking seeds in isosteviol on the dry weight, fresh weight, osmotic adjustment substances, and ROS contents of *Brassica napus* seedlings under salt stress and the moderation effect of Na^+^, K^+^, and Cl^−^ absorption and distribution in seedling tissues. We conducted qualitative analysis on the Fourier transform infrared spectroscopy (FTIR) spectrum of the leaf tissue of rapeseed seedlings to analyze the function and mechanism of isosteviol in the plant salt stress response. This study provides a scientific basis for the investigation of the function of novel plant growth regulatory substances on plant growth and development and for the inducing of defense mechanisms under salt stress, as well as the development and utilization of salinized soil.

## 2. Results

### 2.1. Biomass of Seedlings

**Figure 1:**
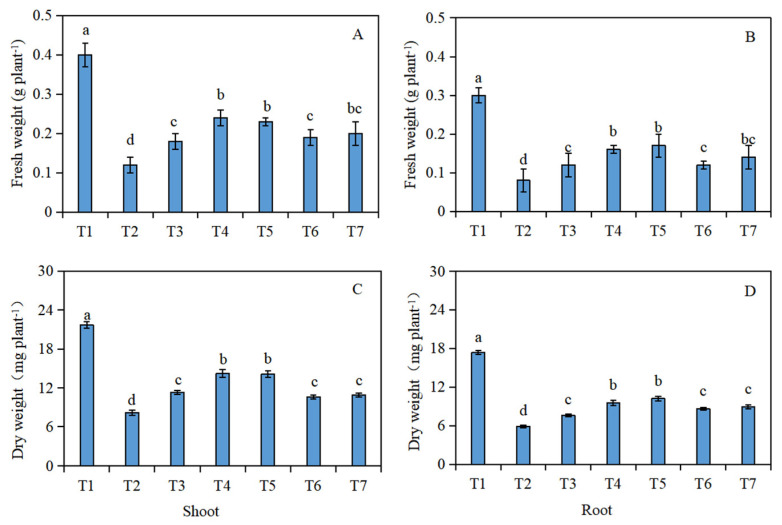
Effect of isosteviol on the biomass of *Brassica napus* seedlings under salt stress. T1: control group; T2: NaCl; T3: NaCl + isosteviol (10^−10^); T4: NaCl + isosteviol (10^−9^); T5: NaCl + isosteviol (10^−8^); T6: NaCl + isosteviol (10^−7^); T7: NaCl + isosteviol (10^−6^). Data are presented as the mean ± S.D. (*n* = 5 in each group). Different lowercase letters indicate significant differences between different treatments (*p* ≤ 0.05). (**A**) Fresh weight of shoot; (**B**) fresh weight of root; (**C**) dry weight of shoot; (**D**) dry weight of root.

The fresh weight (Figure 1A,B) and dry weight (Figure 1C,D) of the *Brassica napus* seedlings were significantly reduced under salt stress (T2), compared with the control (T1). In addition, compared with the salt stress (T2), the fresh and dry weight of the shoots and roots of the *Brassica napus* seedlings significantly increased after soaking with isosteviol. Among them, the T4 treatment group showed the most significant increase in the fresh and dry weight of the seedling shoots, which increased by 1 and 0.7 times, respectively. The T5 treatment group showed the most significant increase in the fresh and dry weight of the seedling roots, which increased by 1.1 and 0.7 times, respectively.

### 2.2. Contents of Osmotic Substances in the Shoots and Roots

#### 2.2.1. Glycine Betaine Contents in the Shoots and Roots

**Figure 2:**
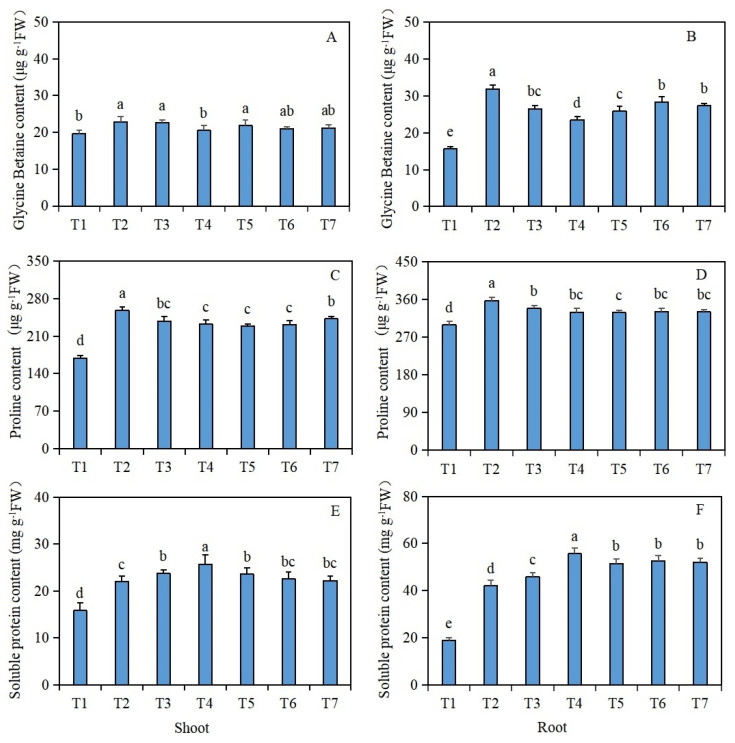
Effects of isosteviol on the osmotic substance contents in the tissues of *Brassica napus* seedlings under salt stress. T1: control group; T2: NaCl; T3: NaCl + isosteviol (10^−10^); T4: NaCl + isosteviol (10^−9^); T5: NaCl + isosteviol (10^−8^); T6: NaCl + isosteviol (10^−7^); T7: NaCl + isosteviol (10^−6^); FW: fresh weight. Data are presented as the mean ± S.D. (*n* = 5 in each group). Different lowercase letters indicate significant differences between different treatments (*p* ≤ 0.05). (**A**) Glycine betaine content of shoot; (**B**) glycine betaine content of root; (**C**) proline content of shoot; (**D**) proline content of root; (**E**) soluble protein content of shoot; (**F**) soluble protein content of root.

The glycine betaine content in the shoots and roots of the *Brassica napus* seedlings was significantly increased under salt stress (T2), compared with the control (T1) (Figure 2A,B). However, compared with salt stress (T2) alone, after seed soaking in isosteviol, the T4 treatment group significantly reduced the glycine betaine content in the shoots of *Brassica napus* seedlings by 10.5%, while the remaining treatment groups had no significant change. Compared with salt stress (T2), after seed soaking in isosteviol, all the treatment groups significantly reduced the glycine betaine content in the roots of *Brassica napus* seedlings, in which the T4 treatment group decreased significantly, with a reduction of 26.3%. 

#### 2.2.2. Proline Content in the Shoots and Roots

The proline content in the shoots and roots of the *Brassica napus* seedlings was significantly increased under salt stress (T2), compared with the control (T1) (Figure 2C,D). However, compared with salt stress (T2) alone, after the seed soaking treatment, all the treatment groups significantly reduced the proline content in the shoots and roots of the *Brassica napus* seedlings, with the T5 treatment group having the most significant reduction. The proline contents of the shoots and roots decreased by 11.2% and 8.1%, respectively. 

#### 2.2.3. Soluble Protein Contents in the Shoots and Roots

The soluble protein content in the shoots and roots of the *Brassica napus* seedlings was significantly increased under salt stress (T2), compared with the control (T1) (Figure 2E,F). However, compared with salt stress (T2) alone, after the seed soaking treatment, all the treatment groups, except for the T6 and T7 treatment groups, significantly increased the soluble protein content in the shoots of the *Brassica napus* seedlings, with the T4 treatment group showing the most significant increase of 16.8%. Compared with salt stress (T2) alone, after the isosteviol seed soaking treatment, all the treatment groups significantly increased the soluble protein content of the roots of the *Brassica napus* seedlings, with the T4 treatment group showing the most significant increase of 32.4%. 

### 2.3. Na^+^, K^+^, Cl^−^ Contents in the Shoots and Roots

Under salt stress, the effects of exogenous isosteviol seed soaking on the Na^+^, K^+^, and Cl^−^ contents of the *Brassica napus* seedlings are shown in Table 1. Compared with the control (T1), under salt stress alone (T2), the Na^+^ and Cl^−^ contents in the shoot and root tissues of the *Brassica napus* seedlings were significantly increased, and the K^+^ contents in the shoot and root tissues of the *Brassica napus* seedlings were significantly reduced. However, compared with salt stress (T2), all the isosteviol treatment groups significantly decreased the Na^+^ and Cl^−^ contents in the shoot and root tissues of the *Brassica napus* seedlings after seed soaking treatment. The T4 and T7 treatment groups reduced the Na^+^ in the shoot and root tissues of the *Brassica napus* seedlings by 22.2% and 34.0%, respectively. The T6 and T7 treatment groups showed the most significant reduction in Cl^−^ in the shoot and root tissues of the *Brassica napus* seedlings, with reductions of 28.6% and 55.5%, respectively. Compared with salt stress (T2), all the treatment groups significantly increased the K^+^ content in the shoot tissues of the *Brassica napus* seedlings after soaking with isosteviol. Except for the T3 treatment group, all the other treatment groups significantly increased the K^+^ content in the root tissues of the *Brassica napus* seedlings. The increase in K^+^ content in the shoot and root tissues of the T6 and T5 treatment groups was the most significant, with increases of 1.6 and 0.6 times, respectively.

Under salt stress, after soaking the seeds in the same concentration of isosteviol, the Na^+^ content in the shoots of the *Brassica napus* seedlings was always lower than that in the roots, while the K^+^ content in the shoots of the *Brassica napus* seedlings was significantly higher than that in the roots. This indicates that under salt stress, the Na^+^ absorbed by the *Brassica napus* seedlings mainly accumulates in the roots, and less Na^+^ is transported to the shoots. More of the K^+^ absorbed by the *Brassica napus* seedlings was retained in the roots. It is speculated that this may be an important mechanism for *Brassica napus* seedlings to relieve Na^+^ toxicity.

**Figure 3:**
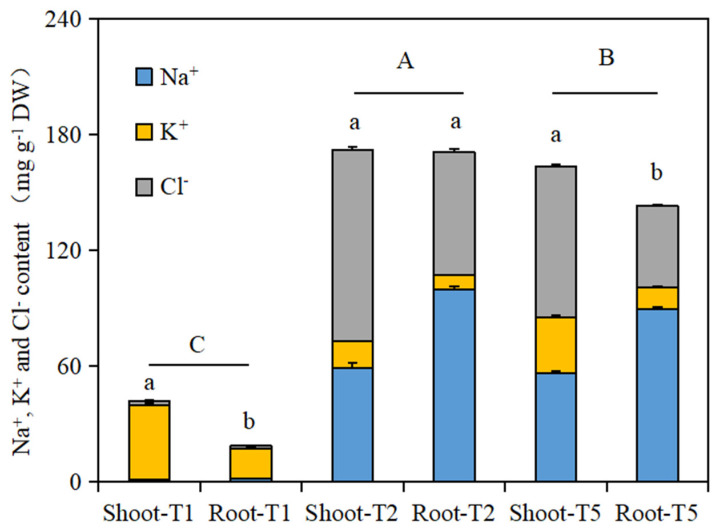
Effects of isosteviol on Na^+^, K^+^, and Cl^−^ contents in different tissues of *Brassica napus* seedlings under salt stress. T1: control group; T2: NaCl; T5: NaCl + isosteviol (10^−8^); DW: dry weight. Data are presented as the mean ± S.D. (*n* = 5 in each group). Different lowercase letters represent significant differences in the total amount of the three ions within shoots and roots in the same treatment group (*p* ≤ 0.05), and different capital letters represent significant differences in the total amounts of the three ions among the different treatment groups (*p* ≤ 0.05).

As shown in Figure 3, the total amounts of the three ions in the shoot and root tissues of the *Brassica napus* seedlings in the salt stress treatment group (T2) and the isosteviol seed soaking treatment group (T5) were significantly higher than those in the control group (T1). There was a significant difference in the total amount of the three ions in the shoot and root tissues of the *Brassica napus* seedlings in the T1 and T5 treatment groups. In addition, the total amount of the three ions in the shoot and root tissues of the *Brassica napus* seedlings in the isosteviol seed soaking treatment group (T5) was significantly lower than that in the salt stress treatment group (T2). This shows that after seed soaking treatment, isosteviol can significantly reduce the uptake of Na^+^ by seedling tissues and can reduce the damage to plant seedlings caused by salt stress.

### 2.4. ROS Contents in the Shoots and Roots

**Figure 4:**
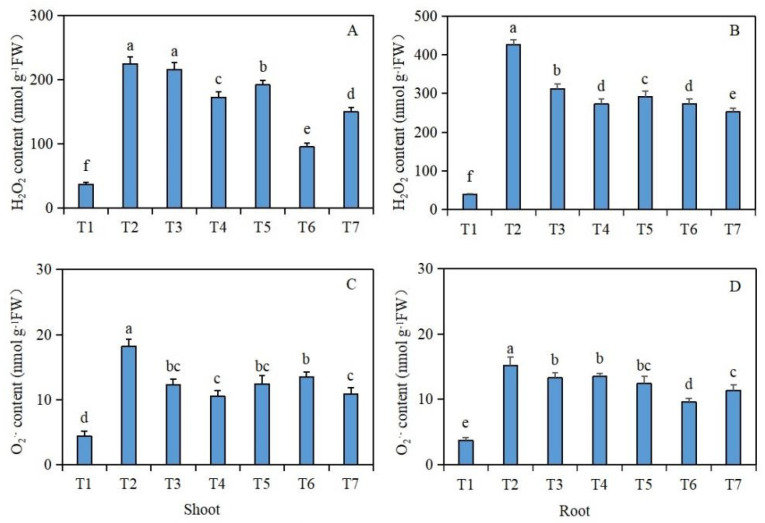
Effect of exogenous isosteviol on the reactive oxygen species (ROS) content of *Brassica napus* seedlings under salt stress. T1: control group; T2: NaCl; T3: NaCl + isosteviol (10^−10^); T4: NaCl + isosteviol (10^−9^); T5: NaCl + isosteviol (10^−8^); T6: NaCl + isosteviol (10^−7^); T7: NaCl + isosteviol (10^−6^); FW: fresh weight. Data are presented as the mean ± S.D. (*n* = 5 in each group). Different lowercase letters indicate significant differences between different treatments (*p* ≤ 0.05). (**A**) H_2_O_2_ content of shoot; (**B**) H_2_O_2_ content of root; (**C**) O_2_^·−^ content of shoot; (**D**) O_2_^·−^ content of root.

Under salt stress, the effects of exogenous isosteviol seed soaking on the H_2_O_2_ and O_2_^·−^ contents of the *Brassica napus* seedlings are shown in Figure 4. Compared with the control group (T1), under salt stress alone (T2), the H_2_O_2_ and O_2_^·−^ contents of the shoot and root tissues of the *Brassica napus* seedlings were significantly increased. However, compared with the salt stress treatment group (T2) alone, after the seed soaking treatment in isosteviol, all the treatment groups except for the T3 treatment group significantly reduced the H_2_O_2_ content in the shoot tissues of the *Brassica napus* seedlings, whereas the T6 treatment group reduced the H_2_O_2_ content most significantly by 57.7% (Figure 4A). In addition, all the isosteviol seed soaking treatment groups significantly reduced the O_2_^·−^ content in the shoot tissues of the *Brassica napus* seedlings, with the T4 treatment group having the most significant reduction, reducing the O_2_^·−^ content by 42.3% (Figure 4C). Compared with the salt stress treatment group (T2) alone, after seed soaking in isosteviol, all the treatment groups significantly reduced the H_2_O_2_ and O_2_^·−^ contents in the root tissues of the *Brassica napus* seedlings, in which the T7 and T6 treatment groups showed the most significant reductions in the H_2_O_2_ and O_2_^·−^ contents in the root tissues of the *Brassica napus* seedlings, with decreases of 40.8% (Figure 4B) and 36.8% (Figure 4D), respectively. These results suggest that under salt stress, the production of ROS in tissues of *Brassica napus* seedlings significantly increases, while isosteviol at an appropriate concentration regulates the antioxidant system in the body, removes redundant ROS, and maintains the dynamic balance of ROS in the body.

### 2.5. Spectroscopy Analysis of Seedling Tissues

**Figure 5:**
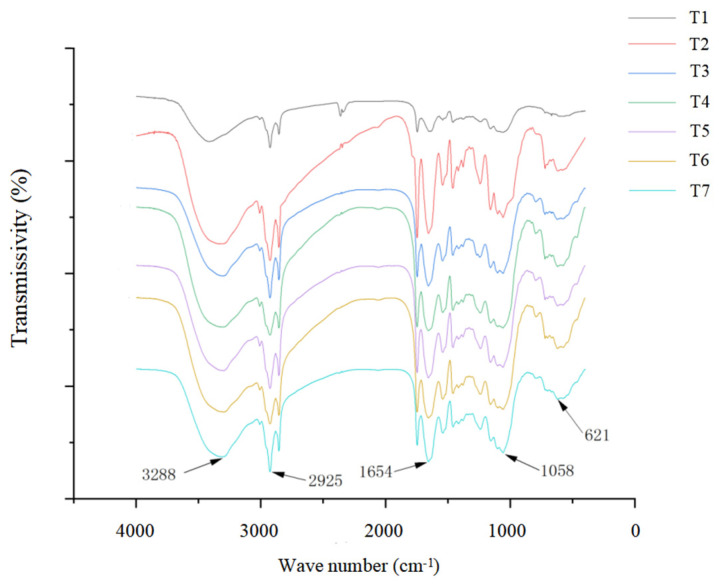
Effect of isosteviol on Fourier transform infrared spectroscopy (FTIR) of *Brassica napus* seedling tissues under salt stress. T1: control group; T2: NaCl; T3: NaCl + isosteviol (10^−10^); T4: NaCl + isosteviol (10^−9^); T5: NaCl + isosteviol (10^−8^); T6: NaCl + isosteviol (10^−7^); T7: NaCl + isosteviol (10^−6^).

Figure 5 shows that, compared with the control group (T1), each characteristic peak did not shift, and no new characteristic peaks appeared in the salt stress treatment group or the isosteviol treatment groups with different concentrations. Only peak intensity changes appeared to a certain extent at positions 621, 1058, 1654, 2925, and 3288 cm^−1^. These results indicated that under salt stress, the main material components of the *Brassica napus* seedlings did not change significantly, but their contents changed. Compared with the control group (T1), in the salt stress alone group (T2), the absorbance values of each characteristic peak of the *Brassica napus* seedling tissue were significantly increased at positions 621, 1058, 1654, 2925, and 3288 cm^−1^. After the seed soaking treatment in isosteviol, the absorbance value of each characteristic peak of the *Brassica napus* seedling tissue significantly decreased and was close to that of the control group (T1).

The characteristic peaks of the infrared spectra of the *Brassica napus* tissues were mainly caused by carbohydrates, proteins, lipids, and nucleic acids. The peaks of the carbohydrates in the tissues mainly appeared between 789 and 1240 cm^−1^, with the characteristic peak at approximately 1053 cm^−1^ being caused by the stretching vibration of carbohydrate C-O and the characteristic peak of approximately 789 cm^−1^ being caused by the external bending vibration of the unsaturated carbon hydrogen in carbohydrates. The protein peaks in the tissues mainly appeared between 1658 and 1415 cm^−1^, with the characteristic peak at approximately 1658 cm^−1^ being caused by protein amide I banded with C=O stretching vibration and the characteristic peak at approximately 1544 cm^−1^ being caused by protein amide II banded with N-H and C-N absorbance peaks; the characteristic peak at approximately 1415 cm^−1^ was caused by the C-O stretching vibration of the protein amide III band. The peaks of the lipids in the tissues mainly appeared between 2925 and 1658 cm^−1^, in which the characteristic peak at approximately 2925 cm^−1^ was caused by the asymmetric stretching vibration of CH_2_; the characteristic peak at approximately 1745 cm^−1^ was caused by the stretching vibration of saturated ester C=O; and the characteristic peak at approximately 1658 cm^−1^ was caused by the stretching vibration of unsaturated ester C=O. The nucleic acid peaks in the tissues mainly appeared between 1240 and 1053 cm^−1^. The characteristic peak at approximately 1240 cm^−1^ was caused by the asymmetric stretching motion of the phosphate group, while the characteristic peak at approximately 1053 cm^−1^ was caused by the symmetric stretching vibration of the phosphate group. Spectroscopy analysis showed that compared with the control group (T1), salt stress (T2) increased the absorbance values of the carbohydrates, proteins, lipids, and nucleic acids, indicating structural damage to the plasma membrane and cell wall. The spectra of the isosteviol seed soaking group (T3–T7) and the control group (T1) were almost the same.

### 2.6. Correlation Analysis of Physiological Indicators

**Figure 6:**
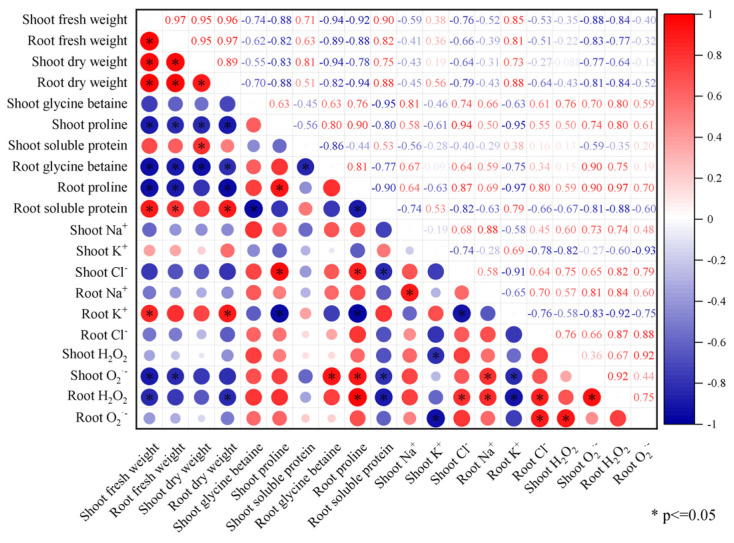
Correlation analysis (T2–T7 treatment groups) of the effects of exogenous isosteviol on the physiological indicators of *Brassica napus* seedlings under salt stress. *: Indicates a significant correlation at the 0.05 level (*p* ≤ 0.05).

Correlation analysis showed that the shoot biomass (fresh and dry weight) of the *Brassica napus* seedlings was significantly negatively correlated with the content of proline and O_2_^·−^ in the shoot (*p* ≤ 0.05) (Figure 6). The root biomass (fresh and dry weight) of the *Brassica napus* seedlings was significantly negatively correlated with the content of glycine betaine, proline, and H_2_O_2_ in the root and significantly positively correlated with the content of soluble protein and K^+^ in the root (*p* ≤ 0.05). The H_2_O_2_ content in the root of the *Brassica napus* seedlings was significantly positively correlated with the Na^+^ and Cl^−^ content in the root and significantly negatively correlated with the K^+^ content in the root (*p* ≤ 0.05). The H_2_O_2_ content in the shoot of the *Brassica napus* seedlings was significantly negatively correlated with K^+^ in the shoot (*p* ≤ 0.05).

### 2.7. Principal Component Analysis

**Figure 7:**
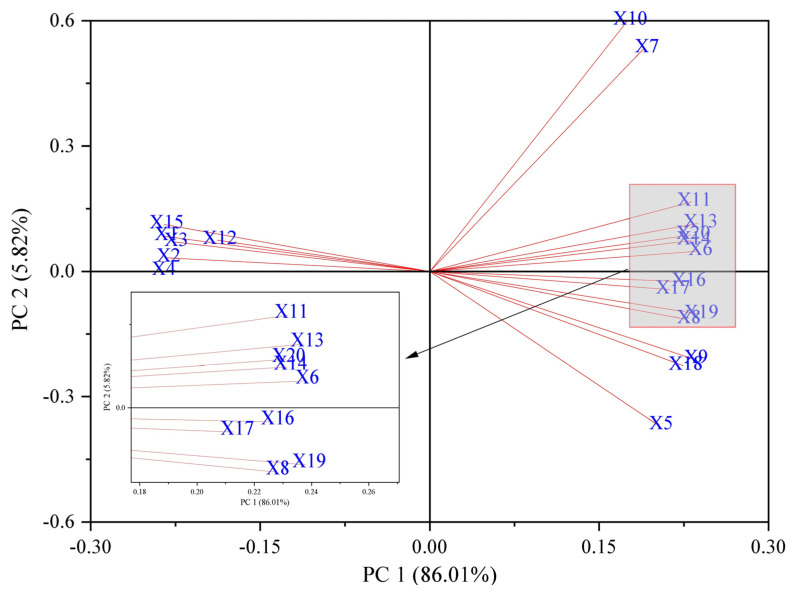
Principal component analysis of physiological indicators of *Brassica napus* seedlings treated with isosteviol under salt stress. X1: shoot fresh weight; X2: root fresh weight; X3: shoot dry weight; X4: root dry weight; X5: shoot glycine betaine; X6: shoot proline; X7: shoot soluble protein; X8: root glycine betaine; X9: root proline; X10: root soluble protein; X11: shoot Na^+^; X12: shoot K^+^; X13: shoot Cl^−^; X14: root Na^+^; X15: root K^+^; X16: root Cl^−^; X17: shoot H_2_O_2_; X18: shoot O_2_^·−^; X19: root H_2_O_2_; X20: root O_2_^·−^.

Principal component analysis (PCA includes all treatment groups of data) was used to evaluate 20 indicators of the physiological changes in the *Brassica napus* seedlings under salt stress by soaking seeds in isosteviol (Figure 7). The results showed that the cumulative contribution rate of PCA1 and PCA2 was 91.83%, which reflected the effect of seed soaking in isosterviol on the physiological and biochemical indicators of the *Brassica napus* seedlings under salt stress. The eigenvalue of PCA1 was 17.20, and the contribution rate was 86.01%. Among them, the indicators with larger positive eigenvalues included the contents of proline, Na^+^, Cl^−^, H_2_O_2_, and O_2_^·−^. The indicators with larger absolute values of negative characteristics included fresh weight, dry weight, and K^+^ content. This shows that when the PCA1 value increases, the values of the osmotic substance and ROS of the *Brassica napus* seedlings also increase under salt stress, while the values of the biomass and K^+^ content indicators that are beneficial for the growth of rapeseed seedlings decrease accordingly. The eigenvalue of PCA2 was 1.16, and the contribution rate was 5.82%. The indicator with a large positive eigenvalue was the soluble protein content, while the glycine betaine content in the shoots of the *Brassica napus* seedlings was highly negatively correlated.

## 3. Discussion

### 3.1. Biomass

Plant seeds are the most sensitive to changes in the external environment during the germination stage and the early growth stage of the seedlings; thus, these are the most critical stages in the entire life history. Salt stress is a common abiotic stress to crops and has become a serious environmental problem that affects plant growth and development. Recently, many studies have shown that plant seed germination is not significantly affected by low concentrations of salt stress, while higher concentrations of salt stress inhibit plant seed germination and the growth and development of seedlings [19,20]. Ren et al. (2020) found that 100 mM NaCl stress significantly inhibited the development of pak choi (*Brassica chinensis* L.) seed germination vigor, germination index, vigor index, and growth of the radicle and embryo [21]. Another study showed that 80 mM NaCl affected the physiological and biochemical indicators of eggplant plants, inhibited the growth rate of eggplants, and caused a decrease in the fruit yield. On the other hand, the exogenous application of indole-3-acetic acid significantly increased the biomass and fruit yield of eggplant plants, overcoming the harmful effects of salt stress [22]. This study found that under 140 mM NaCl stress, the fresh weight and dry weight of the *Brassica napus* seedlings significantly decreased; however, after soaking the seeds with isosteviol, the T4 treatment group showed the most significant increase in the fresh and dry weight of the seedling shoots, while the T5 treatment group showed the most significant increase in the fresh and dry weight of the seedling roots. This may be related to the organic compounds synthesized by *Brassica napus* seedlings through photosynthesis, as well as the differences in respiration caused by salt stress in *Brassica napus* seedlings. This indicates that soaking seeds with isosteviol is beneficial for alleviating the toxicity of salt stress on the growth of *Brassica napus* seedlings. Han et al. (2023) found that compared with the control group (deionized water treatment group), under salt stress the morphological indicators and biomass of wheat seedlings were significantly reduced. However, the reduction in biomass was alleviated by the combined application of sodium salicylate and folsterine to wheat seedling leaves [23]. In addition, Wang et al. (2022b) found that soaking seeds with isosteviol could increase the chlorophyll content in wheat seedling leaves, which is beneficial for plant photosynthesis and significantly increases the biomass of wheat seedlings under salt stress. This is basically consistent with the results of this study [24].

### 3.2. Osmotic Substance

A high soluble salt content in soil will cause salt damage to plants, including primary salt damage and secondary salt damage [25]. Plants growing in this environment will receive salt stress, and the substances involved in osmotic regulation (proline, glycine betaine, soluble protein, etc.) in plant cells will also accumulate in large quantities, affecting the regulation of plant osmotic potential [26]. Studies have shown that under salt stress, gibberellin treatment at a certain concentration can relieve the effect of salt stress on *Paulownia* seeds and seedlings and improve the salt tolerance of *Paulownia* [27,28]. Wang et al. (2022b) found that under salt stress, Lübaonuo millet (*Broomcorn Millet*) seedling root soluble protein and free proline contents were significantly increased. After gibberellin application, the soluble protein content in the seedling roots was significantly lower than that under single-salt stress treatment and was the same as that of the control [29]. Studies have shown that chickpea plants under cadmium pollution alone exhibited higher levels of electrolytic leakage, H_2_O_2_, and MDA, while osmotic protectants such as proline and glycine betaine in the leaves were also significantly increased. After foliar spraying with (10^−6^ M) gibberellin (GA_3_), the accumulation of ROS, MDA, and electrolytic leakage could be significantly reduced, while further increasing the content of osmotic substances such as proline and glycine betaine in the plant resulted in higher tolerance to abiotic stress [28]. In addition, Kamran et al. (2021) found that under salt stress (200 mM NaCl), 0.2 mM kinetin and 2 mM calcium could effectively increase the accumulation of osmotic substances such as proline, soluble proteins, and soluble sugars in the tissues of choysum seedlings, while upregulating the activities of superoxide dismutase, peroxidase, catalase and ascorbate peroxidase significantly reduced the generation of ROS, such as hydrogen peroxide and superoxide anion, during seed germination, thereby maximizing the reduced NaCl-induced oxidative damage [30].

This experiment showed that, compared with the control group (T1), under salt stress alone (T2) the contents of glycine betaine, proline, and soluble protein in the shoots and roots of the *Brassica napus* seedlings were significantly increased. However, compared with salt stress alone (T2), after the seed soaking treatment, the glycine betaine and proline contents of the *Brassica napus* seedlings in some treatment groups were significantly decreased. All the treatment groups significantly increased the soluble protein content in the shoots (except for the T6 and T7 treatment groups) and roots of the *Brassica napus* seedlings. This shows that after seed soaking, isosteviol can reduce the damage caused by salt stress by regulating the contents of osmotic substances in the tissues of rapeseed seedlings.

### 3.3. Absorption and Distribution of Na^+^, K^+^, and Cl^−^

The growth of seeds is highly susceptible to salt stress. In high-salt environments, plants accumulate a large amount of Na^+^ intracellularly. High concentrations of Na^+^ exert the dual effects of osmotic stress and ion toxicity on the body. Physiological drought caused by salinity reduces the net photosynthetic rate of leaves and eventually leads to the inhibition of the yield [31]. Ion toxicity is damaging to plant organs due to the excessive accumulation of harmful ions [32]. Active Na^+^ efflux, Na^+^ compartmentalization, and reduction in K^+^ loss from the cytoplasm are effective mechanisms to maintain Na^+^/K^+^ balance and improve salt tolerance in plants [33]. Salt-tolerant plants can accumulate most of the Na^+^ in the roots, thereby reducing the contents in the shoots and ensuring the normal growth of plants. In addition, plants can regulate the concentrations of various ions through transporter proteins to increase the content of K^+^ in plants, thereby enhancing the salt resistance of plants [34]. Some studies have shown that under salt stress, the K^+^ contents of the salt-tolerant lines KY-1 and KY-191 of Chinese cabbage-type winter rapeseed were significantly higher than those of the sensitive line NDJ, especially the higher content of K^+^ in the leaves, which could relieve Na^+^ poisoning and maintain good intracellular physiological status [35]. Kamran et al. (2021) found that pretreatment of choysum seeds with 0.2 mM kinetin and 2 mM calcium could offset NaCl-induced ion toxicity by reducing Na^+^ and increasing K^+^ content in the tissue, maintaining a balance of the Na/K ratio in the embryonic roots and buds of choysum seeds [30].

In this experiment, under salt stress, after soaking the seeds in the same concentration of isosteviol, the Na^+^ content in the shoot tissues of the *Brassica napus* seedlings was always lower than that in the roots, while more K^+^ from the *Brassica napus* seeds was transported to the shoots. This indicated that under salt stress, the Na^+^ absorbed by the *Brassica napus* seedlings mainly accumulated in the roots, and less Na^+^ was transported to the shoots, while more K^+^ absorbed by the *Brassica napus* seedlings was retained in the shoots. It is speculated that this may be an important mechanism for *Brassica napus* seedlings to relieve Na^+^ toxicity. Studies have shown that under salt stress, plant cell membrane permeability increases and ion selectivity decreases. A large amount of Na^+^ and Cl^−^ enter plant cells. Due to the similar hydration radius of K^+^ and Na^+^, excessive Na^+^ can replace K^+^, leading to K^+^ leakage, which is one of the important characteristics of salt damage [36]. In addition, calcium serves as a protective agent for the cell membrane, bridging the phosphate and phosphate lipids on the membrane surface, as well as the hydroxyl groups of proteins, to maintain the stability of the cytoplasmic membrane, chloroplast membrane, and leaf vesicle membrane under salt stress [37]. Chandran et al. (2023) found that under salt stress, the Na^+^ content in wild-type *Arabidopsis thaliana* plants significantly increased, while the K^+^ content significantly decreased. CaCl_2_ treatment significantly increased the intracellular K^+^ retention rate of wild-type seedlings. Ca^2+^ plays a key role in mediating salt stress response during germination by regulating genes that maintain Na^+^ and K^+^ homeostasis [38]. In addition, under salt stress, the total amount of Na^+^, K^+^, and Cl^−^ in the *Brassica napus* seedlings was significantly higher than that in the control group (T1). After soaking in isosteviol, the total amount of the three ions in the tissues of the *Brassica napus* seedlings in the T5 treatment group was significantly lower than that in the salt stress treatment group alone (T2). This shows that after seed soaking treatment, isosteviol can significantly reduce the uptake of Na^+^ by seedling tissues and reduce the damage to plant seedlings caused by salt stress.

### 3.4. Generation of ROS

Adverse stress often forces plants to produce too many ROS (superoxide radicals, hydrogen peroxide, hydroxyl free radicals, etc.), which leads to the excessive accumulation of free radicals, the aggravation of membrane lipid peroxidation, and the inactivation of important intracellular enzymes and proteins. The decomposition of DNA and even DNA unwinding occur, thus jeopardizing plant growth and development [39,40,41]. Some studies have shown that under salt stress, after treating rice seedlings with exogenous gibberellin, gibberellin could reduce the MDA content and O_2_^·−^ formation in leaves [42,43]. Yan et al. (2021) found that the addition of exogenous melatonin could improve the activities of antioxidant enzymes and reduce the content of ROS in rice seedlings under salt stress. When the concentration of melatonin increases, the content of H_2_O_2_ and O_2_^·−^ significantly decreases, effectively alleviating the oxidative damage to rice seed germination and seedlings caused by salt stress [44]. Wang et al. (2022b) found that under 100 mM NaCl stress, seed soaking in isosteviol could significantly increase the antioxidant enzyme activities of wheat seedlings, reduce the production of MDA, and relieve the stress effect of salt stress on the growth of early wheat seedlings, thereby improving the stress tolerance of wheat [24].

In this experiment, compared with the control group (T1), under salt stress alone (T2) the H_2_O_2_ and O_2_^·−^ contents in the shoots and roots of the *Brassica napus* seedlings were significantly increased. After seed soaking in isosteviol, compared with the salt stress treatment group (T2) alone, all the isosteviol seed soaking treatment groups had significantly reduced H_2_O_2_ (except the T3 treatment group) and O_2_^·−^ contents in the shoot tissues of the *Brassica napus* seedlings. In addition, compared with the salt stress treatment group (T2) alone, all the isosteviol treatment groups had significantly decreased H_2_O_2_ and O_2_^·−^ contents in the root tissues of the *Brassica napus* seedlings. This shows that under salt stress, the production of ROS in *Brassica napus* seedling tissues is significantly increased and that isosteviol at an appropriate concentration can remove excess ROS and maintain the dynamic balance of ROS by regulating the antioxidant system in the body.

FTIR is a structural analysis technique based on functional groups and polar bond vibrations in compounds that can help determine which functional groups are present in molecules and reflect the degree of differences in plant chemical composition from the infrared spectra of different samples [45]. Biomacromolecules such as nucleic acids, lipids, and carbohydrates in plants have characteristic functional groups and unique molecular vibration modes. Analyzing the absorption position, width, and intensity of infrared spectra is helpful in understanding the composition and structural characteristics of organic molecular functional groups in plants. Ye et al. (2020) showed by infrared spectroscopy that compared with the control, cadmium treatment increased the absorbance values of amino groups, hydroxyl groups, cellulose, and epoxide in rice seedlings, indicating that there was structural damage in the biomass membrane and cell wall. After treatment with 160 mg L^−1^ of calcium ions, the infrared spectrum of rice seedlings was basically the same as that of the control group [46]. In this experiment, Fourier infrared spectroscopy data analysis showed that, compared with the control group (T1), salt stress treatment increased the absorbance values of the carbohydrates, proteins, lipids, nucleic acids, and other substances. The spectra of the isosteviol seed soaking group and the control group (T1) were almost the same. This indicates that *Brassica napus* seedlings act on cell walls and membranes with a large amount of carbohydrates, proteins, lipids, and other substances in response to salt stress environments. Studies have shown that under aluminum toxicity stress, the material metabolism of rape seedling leaves undergoes significant changes. A large amount of proteins, carbohydrates, and lipids in the cells act on the cell wall and membrane to resist and adapt to aluminum toxicity stress, while the addition of exogenous boron significantly reduces the content of these substances [47]. Recent studies have found that seed soaking at an appropriate concentration of isosteviol (10^−9^~10^−8^ M) could effectively relieve the growth inhibition of wheat seedlings by the heavy metal chromium, thereby improving the stress tolerance of wheat [18].

Correlation analysis showed that the H_2_O_2_ content in the root of the *Brassica napus* seedlings was significantly positively correlated with the Na^+^ and Cl^−^ content in the root and significantly negatively correlated with the K^+^ content in the root (*p* ≤ 0.05). The H_2_O_2_ content in the shoot of the *Brassica napus* seedlings was significantly negatively correlated with K^+^ in the shoot (*p* ≤ 0.05). This indicated that under salt stress, a large amount of Na^+^ in the solution could enter the cell through the root cytoplasmic membrane. Some Na^+^ accumulates in the roots, while the rest is loaded into the xylem and transported upwards with transpiration. Excessive accumulation in the leaves alters the ion homeostasis of the plant, leading to potential water stress and Na^+^ toxicity, causing a state of peroxidation in the plant’s internal environment. However, the activity of the antioxidant enzyme system in the cells still could not completely eliminate ROS. Ultimately, it causes membrane leakage and oxidative damage to the body. After seed soaking treatment, isosteviol could significantly reduce the absorption of Na^+^ by the seedling tissues, increase the K^+^ content, and reduce the salt stress damage to the plant seedlings. In addition, the results of principal component analysis showed that under salt stress, the contents of proline, Na^+^, Cl^−^, H_2_O_2_, and O_2_^·−^ in the *Brassica napus* plants were significantly increased, while the indicators that were beneficial for *Brassica napus* growth, such as fresh weight, dry weight, and K^+^ content, decreased accordingly.

## 4. Conclusions

In summary, soaking seeds in an appropriate concentration of isosteviol can effectively relieve the effect of the injury of salt stress on the growth of *Brassica napus* seedlings. Among them, treatment with 10^−9^~10^−8^ M isosteviol effectively increased the fresh weight, dry weight and soluble protein content of the *Brassica napus* seedlings and reduced the content of proline, glycine betaine, ROS, and Na^+^ in the seedlings. By regulating the production of osmotic substances and ROS and reducing the oxidative damage to *Brassica napus* seedlings caused by salt stress, the integrity of the biomass membranes in *Brassica napus* seedlings is protected, and their salt resistance is improved.

## 5. Materials and Methods

### 5.1. Test Materials

The rape plants utilized in our experiments were *Brassica napus* Qinyou 2, purchased from Henan Botao Seed Industry Co., Ltd, Zhengzhou, China. Qinyou 2 is a hybrid rapeseed variety of winter *Brassica napus* with the characteristics of early maturity, wide adaptability, and strong stress resistance.

Isosteviol, whose molecular weight is 318.4504 and molecular formula is C_20_H_30_O_3_, was purchased from Shanghai Yuanye Biotechnology Co., Ltd, Shanghai, China.

### 5.2. Experimental Design

First, the *Brassica napus* seeds were washed with deionized water to remove the shriveled and small seeds. Seeds with plump grains and uniform size were selected, and the mass of 50 seeds was controlled to be between 0.15 and 0.2 g. The *Brassica napus* seeds were soaked in 0.002 g mL^−1^ potassium permanganate solution for 20 min, washed 2–3 times with deionized water and blotted dry. Then, the *Brassica napus* seeds were placed in deionized water (T2) and isosteviol at solution concentrations of 10^−10^ (T3), 10^−9^ (T4), 10^−8^ (T5), 10^−7^ (T6), and 10^−6^ M (T7) for 24 h. Then, the *Brassica napus* seeds were removed and placed into Petri dishes with double layers of filter paper (50 grains per dish). According to the results of the previous test [16], 20 mL of NaCl solution at a concentration of 140 mM was separately added to the above six treatment groups for stress treatment. Seeds soaked in deionized water and stressed without NaCl solution (20 mL of deionized water) were used as the control group (T1), and each group of experiments had 5 replications. The culture medium was changed every day, and the cultures were placed in a constant-temperature (25 °C) light incubator with a light intensity of 2500 lux, a light time of 12 h, and a dark time of 12 h. Germination was terminated when no seeds germinated for 4 consecutive days. After 7 d of culture, an appropriate amount of plant tissues was collected to measure the relevant physiological and biochemical indicators.

### 5.3. Sampling and Sample Analysis

#### 5.3.1. Determination of Seedling Biomass

On the 7th day after sowing, we randomly selected five *Brassica napus* seedlings from each Petri dish and rinsed the surface of the seedlings with deionized water; we weighed them with a scale, recorded the fresh weight of the shoot and the root, packed them into bags, fixed them at 105 °C for 30 min, dried them at 65 °C to a constant weight, and recorded the dry weight with a balance [48].

#### 5.3.2. Determination of Shoot and Root Osmotic Substances

The shoots and roots (0.5 g) were homogenized in ethanol (70%) and centrifuged at 10,000× *g* for 10 min to determine their proline contents. The diluted extract reacted to ninhydrin (1%) in acetic acid (60%) in a water bath (95 °C for 20 min), and absorbance at 520 nm was read by the UV-Vis recording spectrophotometer [49] (WFZ UV-2000, Unico^TM^ Shanghai Instrumentation Co., Shanghai, China).

The glycine betaine content was determined according to the method of Valadez-Bustos et al. (2016) [50]. The shoots and roots (0.1 g) were homogenized with H_2_SO_4_ (1 M) and then centrifuged at 12,000× *g* (25 °C, 15 min). The extract was mixed with KI-I_2_ reagent, stored at 0 °C for 16 h, and centrifuged at 12,000× *g* (0 °C, 15 min). The obtained periodite crystals were diluted in acetone, and the absorbance was read at 365 nm.

The soluble protein content was determined by the Coomassie brilliant blue method [51]. The shoots and roots (0.1 g) were ground into a homogenate with deionized water (5 mL), which was subsequently centrifuged for 10 min at 3000× *g*. One milliliter of the supernatant was transferred to a test tube and diluted 5 times with deionized water (4 mL). One milliliter of the diluted solution was mixed with 5 mL of Coomassie brilliant blue G-250. The absorbance was measured at 595 nm after 2 min with a spectrophotometer.

#### 5.3.3. Determination of Na^+^, K^+^, and Cl^−^ in the Shoots and Roots

The contents of Na^+^, K^+^, and Cl^−^ were determined according to the method of Chakraborty et al. (2016) [52]. Take the whole seedlings from each Petri dish, rinse them with deionized water, separate the shoot and root parts, fix them at 105 °C for 30 min, and dry them to a constant weight at 65 °C. Then, take 1.0 g of the dried shoot and root samples, and use concentrated H_2_SO_4_–H_2_O_2_ wet digestion; the content of Na^+^, K^+^, and Cl^−^ ions in the shoots and roots was measured by atomic absorption spectrometry (Hitachi Z-5000, Hitachi Instruments Co., Ltd., Tokyo, Japan).

#### 5.3.4. Determination of Shoot and Root ROS

The H_2_O_2_ content was determined according to the method of Velikova et al. (2000) [53]. The shoots and roots (0.1 g) were used to prepare a tissue homogenate with 1 mL of 5% (*w*/*v*) trichloroacetic acid. Then, the homogenate was centrifuged (9000× *g*, 15 min, 4 °C). The supernatant was mixed with the reaction mixture (containing 2.5 mM potassium phosphate buffer (pH 7.0) and 500 mM potassium iodide), and the absorption was measured at 390 nm.

The hydroxylamine hydrochloride oxidation reaction method was used to determine the O_2_^·−^ content [54]. The shoots and roots (0.1 g) were used to prepare a tissue homogenate with a 65 mM phosphate buffer (pH 7.8). The homogenate was filtered and centrifuged (9000× *g*, 15 min, 4 °C). A total of 2 mL of the reaction mixture containing 1 mL of supernatant, 0.9 mL of 65 mM potassium phosphate buffer (pH 7.8), and 0.1 mL of 10 mM hydroxylamine hydrochloride was reacted at 25 °C for 30 min. Finally, 1 mL of 17 mM sulfanilic acid and 1 mL of 7 mM α-naphthylamine were added, and the absorption was measured at 530 nm.

#### 5.3.5. Spectroscopy Analysis

The dry sample of rapeseed seedlings to be tested was carefully ground with an agate mortar under an infrared lamp, and the KBr tablet method was used for FTIR [55].

### 5.4. Data Statistics and Analysis

The data were calculated using Microsoft Excel 2007 software version: 12.0.4518.1014. The SPSS statistics 21.0 data processing system was used for the statistical analysis, Origin 2021b was used for drawing, and Duncan’s multiple range test was used for the difference test at the level of *p* ≤ 0.05.

## Figures and Tables

**Table 1:** Effect of exogenous isosteviol on Na^+^, K^+^, and Cl^−^ content in *Brassica napus* seedlings under salt stress.

Treatment	Shoot (mg g^−1^ DW)			Root (mg g^−1^ DW)		
	Na^+^	K^+^	Cl^−^	Na^+^	K^+^	Cl^−^
T1	1.2 ± 0.1 e	38.7 ± 0.8 a	2.1 ± 0.2 e	1.6 ± 0.1 e	15.6 ± 0.7 a	1.5 ± 0.1 f
T2	59.1 ± 2.7 a	13.6 ± 0.4 f	99.3 ± 1.8 a	99.9 ± 1.5 a	7.2 ± 0.3 d	63.8 ± 1.9 a
T3	51.2 ± 1.7 c	16.4 ± 0.6 e	85.1 ± 1.4 b	70.9 ± 0.9 c	10.2 ± 0.5 c	49.6 ± 0.9 b
T4	46.0 ± 1.2 d	16.7 ± 0.5 e	78.0 ± 2.1 c	70.5 ± 1.3 c	10.4 ± 0.4 bc	49.6 ± 1.2 b
T5	56.5 ± 0.9 b	28.8 ± 0.8 c	78.0 ± 1.4 c	89.4 ± 1.4 b	11.2 ± 0.5 b	42.5 ± 0.8 c
T6	48.6 ± 0.7 cd	34.9 ± 1.0 b	70.9 ± 1.5 d	71.2 ± 1.2 c	11.0 ± 0.6 b	35.5 ± 0.7 d
T7	51.2 ± 1.4 c	25.7 ± 0.7 d	85.1 ± 1.9 b	65.9 ± 1.8 d	10.4 ± 0.3 bc	28.4 ± 0.9 e

T1: control group; T2: NaCl; T3: NaCl + isosteviol (10^−10^); T4: NaCl + isosteviol (10^−9^); T5: NaCl + isosteviol (10^−8^); T6: NaCl + isosteviol (10^−7^); T7: NaCl + isosteviol (10^−6^); DW: dry weight. Data are presented as the mean ± S.D. (*n* = 5 in each group). Different lowercase letters indicate significant differences between different treatments (*p* ≤ 0.05).

## Data Availability

The data presented in this study are available within the article and its Supplementary Materials.

## Supplementary Materials

The following supporting information can be downloaded at: https://www.mdpi.com/article/10.3390/plants13020217/s1, Table S1: Effect of isosteviol on the biomass of *Brassica napus* seedlings under salt stress. Table S2: Effects of isosteviol on the osmotic substance contents in the tissues of *Brassica napus* seedlings under salt stress. Table S3: Effect of exogenous isosteviol on the reactive oxygen species (ROS) content of *Brassica napus* seedlings under salt stress.
