# Supplementary material for: Effects of Exogenous Isosteviol on the Physiological Characteristics of Brassica napus Seedlings under Salt Stress
Source: Plants (Basel). 2024 Jan 12;13(2):217. doi: 10.3390/plants13020217 (PMC10819195; doi:10.3390/plants13020217)
Supplement: Supplementary file 1 [file plants-13-00217-s001.zip › plants-2785973-supplementary.pdf]

Supplementary Materials

Table S1. Effect of isosteviol on the biomass of *Brassica napus* seedlings under salt stress.

Treatment	Shoot		Root	
	Fresh Weight (g plant^{-1})	Dry Weight (mg plant^{-1})	Fresh Weight (g plant^{-1})	Dry Weight (mg plant^{-1})
T1	0.40 ± 0.03 a	21.72 ± 0.51 a	0.30 ± 0.02 a	17.41 ± 0.31 a
T2	0.12 ± 0.02 d	8.21 ± 0.39 d	0.08 ± 0.03 d	5.90 ± 0.22 d
T3	0.18 ± 0.02 c	11.32 ± 0.31 c	0.12 ± 0.03 c	7.62 ± 0.23 c
T4	0.24 ± 0.02 b	14.20 ± 0.62 b	0.16 ± 0.01 b	9.53 ± 0.42 bc
T5	0.23 ± 0.01 b	14.11 ± 0.48 b	0.17 ± 0.03 b	10.21 ± 0.41 b
T6	0.19 ± 0.02 c	10.63 ± 0.32 c	0.12 ± 0.01 c	8.59 ± 0.21 b
T7	0.20 ± 0.03 bc	10.92 ± 0.30 c	0.14 ± 0.03 bc	8.93 ± 0.29 bc

T1: control group; T2: NaCl; T3: NaCl + isosteviol (10^{-10}); T4: NaCl + isosteviol (10^{-9}); T5: NaCl + isosteviol (10^{-8}); T6: NaCl + isosteviol (10^{-7}); T7: NaCl + isosteviol (10^{-6}). Data are presented as the mean ± S.D. (n = 5 in each group). Mean values with the same letter presented in the column represent the absence of significant difference ($p \leq 0.05$).

Table S2. Effects of isosteviol on the osmotic substance contents in the tissues of *Brassica napus* seedlings under salt stress.

Treatment	Shoot			Root		
	Glycine Betaine (μg g^{-1} FW)	Proline (μg g^{-1} FW)	Soluble Protein (mg g^{-1} FW)	Glycine Betaine (μg g^{-1} FW)	Proline (μg g^{-1} FW)	Soluble Protein (mg g^{-1} FW)
T1	19.7 ± 0.9 b	169.3 ± 4.7 d	15.8 ± 1.7 d	15.6 ± 0.7 e	299.3 ± 8.9 d	18.8 ± 1.1 e
T2	22.9 ± 1.4 a	257.8 ± 7.1 a	22.0 ± 1.1 c	31.9 ± 1.1 a	357.8 ± 7.4 a	42.0 ± 2.3 d
T3	22.6 ± 0.7 a	238.5 ± 8.8 bc	23.7 ± 0.8 b	26.5 ± 0.8 bc	338.5 ± 6.7 b	45.8 ± 1.8 c
T4	20.5 ± 1.3 b	232.4 ± 7.9 c	25.7 ± 1.9 a	23.5 ± 0.9 d	330.2 ± 9.3 bc	55.6 ± 2.5 a
T5	21.9 ± 1.5 a	228.9 ± 4.4 c	23.6 ± 1.3 b	25.8 ± 1.4 c	328.7 ± 5.5 c	51.5 ± 1.8 b
T6	21.0 ± 0.6 ab	231.5 ± 7.5 c	22.5 ± 1.5 bc	28.3 ± 1.5 b	331.5 ± 7.6 bc	52.5 ± 2.4 b
T7	21.2 ± 0.8 ab	242.4 ± 4.6 b	22.2 ± 0.9 bc	27.3 ± 0.6 b	330.4 ± 5.8 bc	52.1 ± 1.5 b

T1: control group; T2: NaCl; T3: NaCl + isosteviol (10^{-10}); T4: NaCl + isosteviol (10^{-9}); T5: NaCl + isosteviol (10^{-8}); T6: NaCl + isosteviol (10^{-7}); T7: NaCl + isosteviol (10^{-6}); FW: fresh weight. Data are presented as the mean ± S.D. (n = 5 in each group). Mean values with the same letter presented in the column represent the absence of significant difference ($p \leq 0.05$).

Table S3. Effect of exogenous isosteviol on the reactive oxygen species (ROS) content of *Brassica napus* seedlings under salt stress.

Treatment	Shoot		Root	
	H_2O_2 (nmol g^{-1} FW)	O_2^{-} (nmol g^{-1} FW)	H_2O_2 (nmol g^{-1} FW)	O_2^{-} (nmol g^{-1} FW)
T1	36.9 ± 2.7 f	4.4 ± 0.7 d	38.9 ± 2.1 f	3.7 ± 0.4 e
T2	224.8 ± 11.1 a	18.2 ± 1.1 a	426.4 ± 12.3 a	15.2 ± 1.3 a
T3	216.1 ± 10.8 a	12.3 ± 0.8 bc	312.3 ± 11.8 b	13.3 ± 0.8 b
T4	172.8 ± 8.9 c	10.5 ± 0.9 c	272.8 ± 13.5 d	13.5 ± 0.5 b
T5	191.8 ± 7.3 b	12.4 ± 1.3 bc	291.8 ± 14.8 c	12.4 ± 1.1 bc
T6	95.2 ± 5.5 e	13.5 ± 0.7 b	273.2 ± 12.4 d	9.6 ± 0.6 d
T7	150.0 ± 6.9 d	10.9 ± 0.9 c	252.3 ± 9.5 e	11.4 ± 0.8 c

T1: control group; T2: NaCl; T3: NaCl + isosteviol (10^{-10}); T4: NaCl + isosteviol (10^{-9}); T5: NaCl + isosteviol (10^{-8}); T6: NaCl + isosteviol (10^{-7}); T7: NaCl + isosteviol (10^{-6}); FW: fresh weight. Data are presented as the mean ± S.D. (n = 5 in each group). Mean values with the same letter presented in the column represent the absence of significant difference ($p \leq 0.05$).
